# Muscle membrane integrity in Duchenne muscular dystrophy: recent advances in copolymer-based muscle membrane stabilizers

**DOI:** 10.1186/s13395-018-0177-7

**Published:** 2018-10-10

**Authors:** Evelyne M. Houang, Yuk Y. Sham, Frank S. Bates, Joseph M. Metzger

**Affiliations:** 1 Department of Integrative Biology and Physiology, University of Minnesota Medical School, 6-125 Jackson Hall, 321 Church Street SE, Minneapolis, MN 55455 USA; 2 University of Minnesota Informatics Institute, MN, USA; 3 Bioinformatics and Computational Biology Program, University of Minnesota, MN, USA; 4 Department of Chemical Engineering and Materials Science, University of Minnesota, MN, USA

**Keywords:** Duchenne muscular dystrophy, Block copolymers, Membrane stabilization

## Abstract

The scientific premise, design, and structure-function analysis of chemical-based muscle membrane stabilizing block copolymers are reviewed here for applications in striated muscle membrane injury. Synthetic block copolymers have a rich history and wide array of applications from industry to biology. Potential for discovery is enabled by a large chemical space for block copolymers, including modifications in block copolymer mass, composition, and molecular architecture. Collectively, this presents an impressive chemical landscape to leverage distinct structure-function outcomes. Of particular relevance to biology and medicine, stabilization of damaged phospholipid membranes using amphiphilic block copolymers, classified as poloxamers or pluronics, has been the subject of increasing scientific inquiry. This review focuses on implementing block copolymers to protect fragile muscle membranes against mechanical stress. The review highlights interventions in Duchenne muscular dystrophy, a fatal disease of progressive muscle deterioration owing to marked instability of the striated muscle membrane. Biophysical and chemical engineering advances are presented that delineate and expand upon current understanding of copolymer-lipid membrane interactions and the mechanism of stabilization. The studies presented here serve to underscore the utility of copolymer discovery leading toward the therapeutic application of block copolymers in Duchenne muscular dystrophy and potentially other biomedical applications in which membrane integrity is compromised.

## Background

**Fig. 1:**
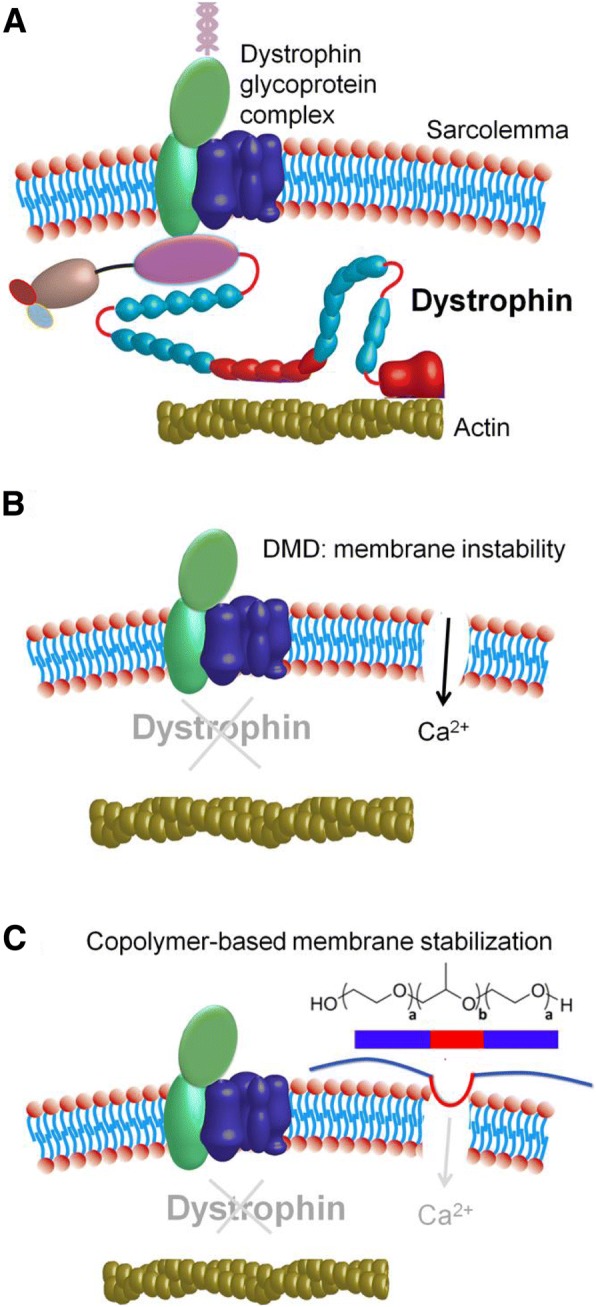
Copolymer-based muscle membrane stabilization of dystrophic muscle. **a** Representation of intact muscle membrane with dystrophin anchoring the DGC to the actin cytoskeleton. **b** Membrane instability caused by the lack of dystrophin leads to pathological increases in intracellular Ca^2+^ concentration. **c** Copolymer stabilization of the damaged membrane via insertion of its hydrophobic PPO block (red) prevents entry of extracellular Ca^2+^ into the cell

All eukaryotic cells are enveloped by a phospholipid bilayer membrane. An enormous literature exists that defines biological cell membrane form and function [1, 2]. Regardless of biological cell type, the cell membrane represents first and last line of defense for ensuring the normal function and ultimately the viability of the cell. Accordingly, multiple cellular processes are present to help ensure the maintenance, repair and protection of the cell membrane. There are numerous excellent expert reviews detailing cell intrinsic mechanisms of membrane integrity and repair [2–17] and mechanistic details on these will not be further elaborated on here. Rather, this review focuses on membrane protection from the perspective of a chemical-based approach to preserve muscle membrane integrity and how this unique cell extrinsic approach could complement cell intrinsic membrane stabilization/repair pathways (Fig. 1). Numerous acquired and inherited diseases comprise, at some level, an etiology involving cell membrane instability. Duchenne muscular dystrophy is the archetype inherited disease of severe membrane fragility and serves as the disease model focal point of this review.

## Duchenne muscular dystrophy: a fatal disease of muscle membrane instability

Duchenne muscular dystrophy (DMD) is an X-linked recessive disease of marked striated muscle deterioration, affecting 1 in 3500–5000 boys [18]. DMD results from the lack of the cytoskeletal protein dystrophin, a protein indispensable for maintaining the structural integrity of the muscle cell membrane [19]. DMD disease onset typically occurs between the ages of 2 and 5 years and is characterized by a delay in achieving childhood motor milestones. DMD presents as a prominent and progressive weakness in limb muscles and postural muscles [18], leading to spinal scoliosis and decrease in exercise capacity. Weakness of the knees and hip extensors are displayed through the Gower’s sign, a maneuver through which the affected child will right himself from a supine position by using his hands and arms to extend the hips and bring the torso to an upright position [20]. Other physical symptoms include reduced muscle bulk, pseudo-hypertrophy, and contractures of the calf muscles and joints [21]. Bone fragility and osteoporosis also contribute to the development of scoliosis [22]. Concurrent with the decline in orthopedic condition is loss of respiratory function brought on by significant diaphragm wasting [23] leading patients to be placed on positive pressure nocturnal ventilation. Loss of ambulation and wheelchair dependency occur by the early teens [24], and DMD patients typically succumb in their 20s due to cardio-respiratory failure [25–28].

DMD patients develop a severe cardiomyopathy, presenting as dilated cardiomyopathy [29], with arrhythmias and eventually heart failure occurring in the second/third decade of life [24]. With increases in patient lifespan, as a result of palliative glucocorticoid treatment and improvements in respiratory care and orthopedic corrections [30, 31], cardiomyopathy is an increasingly important but underappreciated contributor to DMD mortality. It is now evident that cardiomyopathy is present in 90% of DMD patients by age 18 and is confirmed by significant myocardial fibrosis in autopsies [32–35]. Interestingly, the cardiomyopathy usually remains subclinical at early age and cardiac disease progression typically proceeds at a slower rate compared to the skeletal muscle degeneration [36]. The incidence and evolution of cardiomyopathy in Duchenne muscular dystrophy is presumably due to lesser strain on the heart when physical activity is limited once the patient is wheelchair bound.

### Dystrophin

Extensive genetic analysis of DMD patients determined that defects in the dystrophin gene are causal for the disease [19]. The dystrophin gene spans 2.5 Mb of DNA on the X chromosome. Dystrophin’s 79 exons encode a 3685 amino acid cytoskeletal protein localized to the intracellular surface of the muscle membrane [19]. Dystrophin consists of four major functional domains: (1) an actin-binding domain at the N-terminus; (2) a central rod domain consisting of 24 spectrin-like repeats separated by four hinge regions, that has been shown to unfold and give flexibility in response to mechanical stretch [19]; (3) a cysteine-rich domain that interacts with the transmembrane protein β-dystroglycan; and (4) a C-terminal domain, critical for dystrophin’s interaction with other sub-sarcolemmal proteins [37–39]. Detailed structure function-based transgenic animal studies have determined that the domains most critical to DMD pathology are the cysteine-rich domain and the N-terminal domain, and those are directly associated with mechanically linking the extracellular matrix and the cytoskeleton [40].

Dystrophin is part of a large membrane-spanning complex of glycoproteins (dystrophin-glycoprotein complex or DGC) that also include sarcoglycans (α, β, γ, δ), dystroglycans (α and β), dystrobrevins, syntrophins, and sarcospan [38, 39, 41] (Fig. 1a). This dystrophin-associated protein complex is found and enriched at the muscle costamere, a network of proteins that physically connect the extracellular matrix to the cytoskeleton, through the muscle membrane or sarcolemma, and as such orchestrates the lateral force transmission [42–44]. As such, one essential function of dystrophin in striated muscle is to stabilize the muscle membrane against the forces associated with contraction thereby acting as a “molecular shock absorber” or molecular force dampener of the muscle membrane [45, 46]. The importance of dystrophin’s scaffolding support at the membrane is evident in studies showing that dystrophin-deficient muscle fibers where the membrane was experimentally removed show no difference in contractile function compared to normal skeletal muscle fibers, indicating a defect in the membrane-cytoskeleton linkage rather than in the contractile apparatus [47].

### Striated muscle membrane fragility in DMD

Biological membranes are asymmetrical bilayers approximately 5–6 nm thick and comprised of various lipids, including phospholipids, sphingolipids, glycolipids and sterols [48–51]. Phospholipid composition can vary significantly between different cell types and also in disease states [48, 49, 52, 53]. The eukaryotic cell membrane is also typically composed of 20–30% proteins responsible for ion conduction, various signaling pathways, and structural integrity [53]. Irrespective of cell type and function, the primary role of the cellular membrane is to segregate the intracellular milieu from the outside environment to actively preserve intracellular homeostasis. Transmembrane proteins are essential for normal conduction of ions, allowing maintenance of physiological ionic gradients at affordable metabolic cost. Failure to maintain barrier function leads to exhaustion of the metabolic energy of the cell, biochemical arrest, and eventual cellular demise.

The membrane bilayer is held together via hydrophobic effect among phospholipids and their interaction with the surrounding polar solvent environment, involving van der Waals forces, hydrogen bonding, and electrostatic interactions [50, 51, 53, 54, 55, 56, 57]. Membrane constituents are allowed various intra-bilayer motions, including lateral diffusion, rotation of lipids around their major axes, and oscillations [56–58]. Intra-bilayer motion, as well as the degree of packing of bilayer components, is collectively described as “membrane fluidity” [48, 56]. Membrane fluidity is controlled by a number of factors, including lipid composition, sterol enrichment, and temperature. Fluidity is generally assessed using fluorescence polarization methods, electron spin resonance, and other spectroscopic methods [59–62]. Along with membrane fluidity, the structure and composition of the bilayer can be described by parameters such as rigidity, elasticity, and tensile strength, all of which make up the membrane physical property known as plasma membrane order [58, 63]. Various studies have suggested that an optimal level of membrane order is essential for normal myocyte function [57, 64]. Of particular interest to muscle, nicotinic acetylcholine receptors which are present at neuromuscular junctions of muscle cells can be allosterically modulated by surrounding lipids and thus require an optimal membrane microenvironment to retain normal function [65, 66]. Therefore, alterations to the muscle membrane surrounding these receptors, either during mechanical stress or in diseased states, such as in DMD, have important ramifications for ion conductance and thus ultimately affecting action potential generation and propagation during muscle contraction.

From a structural perspective, the lipid bilayer alone is not sufficient to counteract the significant forces placed on the membrane during muscle contraction [67]. Mechanical integrity of the sarcolemma is further supported by key cytoskeletal proteins, including dystrophin, spectrin, and F-actin [68, 69]. Electron microscopy analysis of dystrophic muscle directly shows disruptions in the muscle membrane, termed delta lesions [70, 71]. This discovery led to the theory that the loss of dystrophin and associated proteins at the sarcolemma renders the membrane leaky and the muscle susceptible to contraction-induced injury. Indeed, serum detection of the soluble enzyme creatine kinase as it is released from the injured muscle is a clinical hallmark of the disease [72]. Membrane permeability is further exacerbated by mechanical stress, particularly with lengthening contractions of skeletal muscles such as during downhill walking/running [73]. Lengthening contractions occur when the force applied to the muscle exceeds the force generated by the muscle, resulting in lengthening of the muscle during active contraction. Repetitive lengthening contractions cause significant damage to dystrophic muscle by injuring the membrane and downstream elements, including the EC coupling machinery [74, 75].

In DMD patients, muscle biopsies show active degeneration and regeneration of skeletal muscle fibers and creatine kinase is persistently elevated [18, 27, 76, 77]. Presently, it is unclear the precise nature of membrane disruptions caused by lengthening contractions. However, the release of intracellular enzymes such as creatine kinase and the uptake of large proteins such as albumin and vital dyes like Procion orange [73] and Evans blue [78] into non-necrotic muscle fibers indicate that the membrane disruptions are sufficiently large to permit the transmembrane passage of sizable macromolecules which can be monitored as biomarkers of muscle injury [72]. Lengthening injury is also particularly apparent in the diaphragm which contracts to expand the lungs during breathing. Ventilatory muscles of DMD patients and in animal models have impaired contractility and increased fibrosis [79]. Dystrophin also plays a crucial role in buffering against cardiac myocyte extension [80]. This occurs when the ventricle fills with blood during diastole to cause passive lengthening of myocytes. In dystrophin deficiency, this passive lengthening leads to membrane dysfunction as evidenced by Ca^2+^ entry and uptake of extracellular molecules [80]. Moreover, the consequences of membrane disruptions and increased permeability are intrinsically different between cardiac and skeletal muscle as the process of Ca^2+^ − induced Ca^2+^ release is predominant in the heart [81]. As such, with increases in contractility and larger passive extensions, subsequently more unregulated Ca^2+^ entry into the cell eventually results in terminal contracture of the dystrophic myocyte [80].

### Muscle membrane barrier function is severely disrupted in DMD

Owing to membrane dysfunction, Ca^2+^ homeostasis is perturbed in dystrophic muscle (Fig. 1b). This Ca^2+^ dysregulation is an important component of the pathological processes leading to muscle cell death. Intracellular calcium levels are elevated in both *mdx* skeletal muscle fibers and cardiac myocytes [80, 82–84]. It is still unclear what causes this rise in intracellular Ca^2+^, with some studies suggesting Ca^2+^ entering the cell due to increased membrane permeability or “tears” [80], and other studies showing evidence for the activation of Ca^2+^ leak channels or stretch-activated channels [85]. Regardless of the initial mechanism of entry, this abnormal elevation in Ca^2+^ has consequences to muscle structure and function due to activation of pathological Ca^2+^ sensitive cellular pathways, including activation of the calpain proteases [86] and perturbation of calcium-activated signaling pathways including calmodulin [87], calcineurin [88], and the mitochondrial permeability transition pore [89]. Of importance, activation of calpains by extracellular Ca^2+^ influx leads to cleavage of the transmembrane protein dysferlin, a crucial mediator in the cell intrinsic membrane repair machinery [90, 91]. A pathological rise in cytosolic Ca^2+^ also contributes to membrane damage via activation of phospholipase A2 and promotion of reactive oxygen species (ROS) production by the mitochondria [92]. ROS in turn leads to peroxidation of membrane lipids [93, 94]. Additionally, mitochondrial Ca^2+^ overload promotes irreversible opening of the mitochondrial permeability transition pore, aberration of mitochondrial function and reduction of ATP production leading to cellular energy deprivation and cell death. Oxidative stress and elevated intracellular Ca^2+^ signaling are evident in hearts of *mdx* mice before pathological manifestations of cardiomyopathy, and there is increasing evidence of mitochondrial dysfunction in dystrophic striated muscle [89]. Consequently, maintaining intracellular Ca^2+^ homeostasis by preventing the deleterious influx of extracellular Ca^2+^ is crucial to the survival of dystrophic striated muscle. Moreover, another recent study indicates that Ca^2+^ influx can progressively increase in dystrophic muscle and lead to mitochondrial dysfunction. This, in turn, further compromises the endogenous membrane repair ability of dystrophin-deficient myofibers. This negative feedback loop limits the cell intrinsic membrane repair machinery resulting in exacerbation of muscle deterioration in DMD [95].

### Current DMD therapeutic strategies: cell intrinsic/cell extrinsic strategies

There is no cure for DMD nor an effective treatment clinically demonstrated to halt, prevent, or reverse DMD striated muscle deterioration. Glucocorticoids have been the standard of care for DMD but are accompanied by several adverse effects such as excessive weight gain, behavioral issues, growth retardation, osteoporosis, and impairment of glucose metabolism, all associated with chronic long-term use [30, 96]. Prednisolone and deflazacort are regularly administered soon after diagnosis and have been shown to slow the progression of the disease by improving muscle strength and exercise capacity thereby delaying loss of ambulation and improving both pulmonary and cardiac functions. Several ongoing experimental DMD therapeutics feature gene and cell-based strategies [97, 98], including exon-skipping strategies to restore dystrophin production [99–102]. Exon skipping strategies using small molecules have been shown to ameliorate the severe dystrophic phenotype in both canine and murine DMD models [99, 100, 102–104] while being well tolerated and non-immunogenic. One significant caveat is that this strategy is only applicable to the subset of DMD patients with the corresponding targeted mutation. Additionally to date, most of these approaches have not yet been translated successfully in human patients [105, 106]. One exon skipping treatment, eteplirsen (Sarepta Therapeutics Inc.), has recently been approved by the FDA through its accelerated approval pathway. A clinical trial in a small cohort of DMD patients resulted in a dose-dependent partial restoration of dystrophin production with upregulation of other dystrophin-associated proteins at the membrane, along with some improvement in patient walking ability compared to placebo controls [107, 108]. However, this improvement was only observed in a small subset of the patient group, with dystrophin levels observed to be highly variable among all patients, and a larger clinical trial is currently underway to confirm these results across a larger patient group. Unfortunately, eteplirsen is only targeted to approximately 13% of DMD patients with a mutation amenable to exon 51 skipping [108] leaving a large population of DMD patients currently without treatment options.

Many experimental therapeutic efforts preferentially target dystrophic skeletal muscles, leaving the diseased heart untreated [29]. Skeletal muscle-centric strategies to improve ambulation for DMD patients could lead to increased stress on the untreated dystrophic myocardium as a result of increased cardiac demands [29, 109, 110]. This interplay between the progression of DMD cardiomyopathy and the skeletal myopathy as a pathophysiological load on the heart underscores the importance of a therapeutic strategy to effectively treat all striated muscles. In this context, it is worth considering additional approaches that target the primary defect of DMD: severe muscle membrane fragility. As the primary pathophysiological defect in DMD is the marked susceptibility to contraction-induced membrane stress, and the subsequent muscle damage and degeneration that occurs due to loss of muscle membrane barrier function, a unique therapeutic approach is the use of synthetic membrane stabilizers to prevent muscle damage by directly stabilizing the dystrophin-deficient muscle membrane (Fig. 1c).

## Copolymers as cell extrinsic muscle membrane stabilizers

**Fig. 2:**
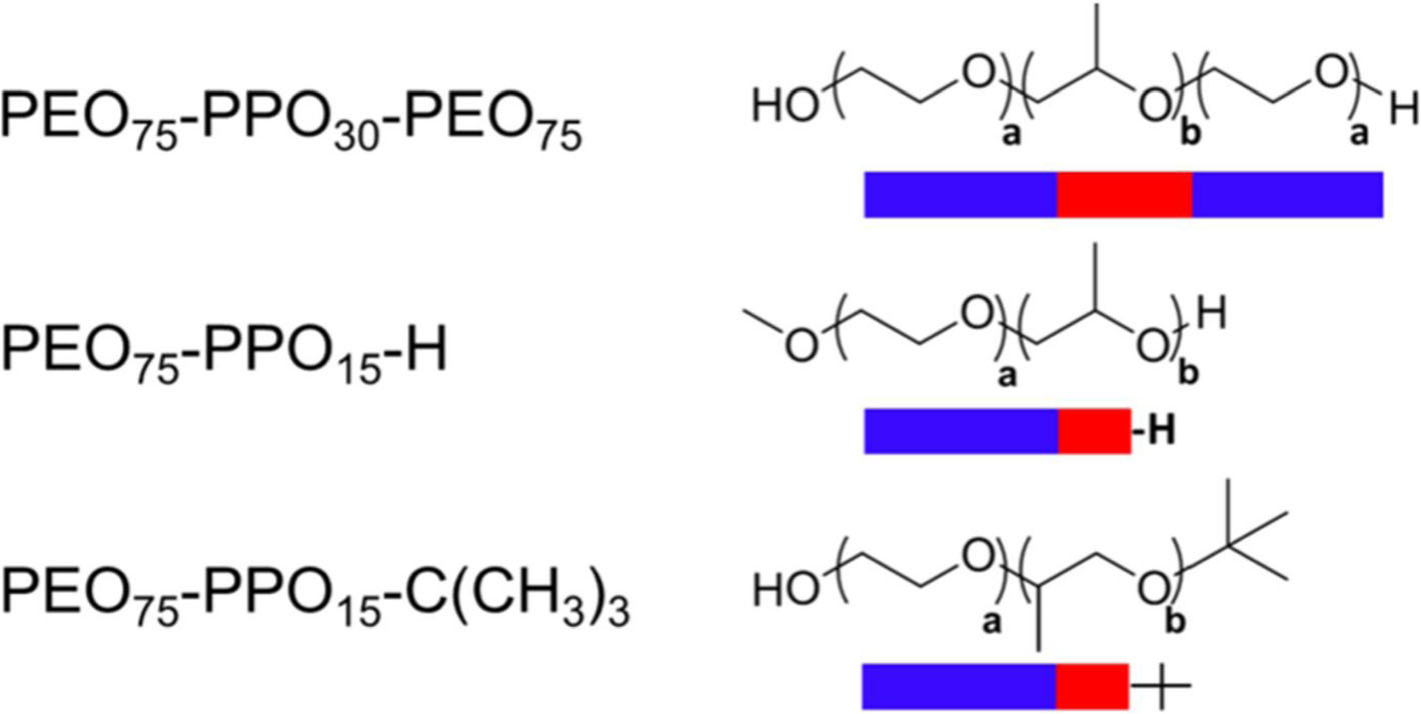
Schematic representation of a triblock and diblock copolymers chemical structures. Chemical structures and representations of the triblock copolymer P188 (PEO_75_–PPO_30_–PEO_75_) and diblocks of P188 (PEO_75_–PPO_15_) with differing end groups (–H and –C(CH_3_)_3_) where a and b represent the number of repeating PEO and PPO group respectively

The triblock copolymer class of membrane-interacting synthetic molecules, known as poloxamers or pluronics, are linear structures comprised of a hydrophobic polypropylene oxide (PPO) core block flanked on both sides by hydrophilic polyethylene oxide (PEO) chains (Fig. 2)(Table 1) [111, 112]. This constitutes the triblock copolymer A-B-A architecture. Poloxamers are non-ionic amphiphiles having topologically distinct hydrophilic and lipophilic components. A wide range of block copolymers with distinct physicochemical properties can be designed by varying the lengths of the PEO and PPO blocks. Poloxamers were the first commercially produced block copolymers, synthesized by Wyandotte Chemical Corporation in the late 1940s for industrial purposes, and now widely found in both industrial and consumer products. Poloxamers span ~ 10–80% wt.% poly(ethylene oxide) and 1000 – 15000 g/mol molecular weight with complex interfacial behavior. Poloxamers have numerous biological applications, including as drug delivery adjuvants, enhancers of drug penetration in the treatment of multiple drug resistant tumors [113, 114], and membrane interacting agents, either as lysis detergents [115–117] or cell membrane stabilizers [80, 118, 119] depending on structure. This latter feature is directly attributed to poloxamers varying affinity for both the surrounding solvent and with the similarly amphiphilic phospholipid membranes [120–123]. An excellent comprehensive review detailing copolymer physical and chemical properties, as well as safety, has been published [124].

**Table 1:** Chemical properties of representative synthetic block copolymers

Architecture	Polymer	PEO^a^	PPO^a^	End group^b^	Mass^c^	PEO%^d^
Triblock copolymer/P188^e^	PEO_75_PPO_30_PEO_75_	150	30	–	8400	80
Triblock copolymer/P338^e^	PEO_140_PPO_44_PEO_140_	280	44	–	8400	84
Triblock copolymer/P331^e^	PEO_7_PPO_54_PEO_7_	14	54	–	3700	26
Diblock copolymer						
	PEO_75_PPO_15_ − H	75	15	−H	4200	80
	PEO_75_PPO_15_ − C4	75	15	−C(CH_3_)_3_	4430	77
Homopolymer	PEO_198_	198	0	–	8700^f^	100

^a^Total number of EO or PO monomer units

^b^Chemical end group at terminal PO

^c^Average molecular weight in g/mol by ^1^H NMR end-group analysis

^d^PEO weight percent to total molecular weight

^e^Manufacturer BASF

^f^Number average molecular weight

In the context of biomedical investigation, poloxamer 188 (P188), with a PPO/PEO ratio of 0.20 and a molecular weight of 8400 Da, is the most widely studied triblock copolymer (Table 1). P188’s earliest reported use was in 1952 as an additive to enhance blood oxygenation [125]. It was found to reduce fat emboli and hemolysis in patients under extended cardiopulmonary bypass [126–128] and as a priming agent in heart-lung bypass [129]. P188 was also incorporated as a wetting agent [130, 131] and an emulsifier for clinically tested drug formulations [132, 133] as well as used as a solubilizing agent of perfluorochemicals which have significant O_2_ carrying capacity to create an emulsion used as an artificial blood substitute [134]. P188 functions as a rheological agent to reduce blood viscosity and platelet aggregation [135–138]. It was also reported that P188 reduces membrane fluidity and improves cell survivability during shear stress in HB-32 hybridoma cell lines, presumably through direct membrane interaction [61]. P188 was subsequently widely deployed as a shear protective agent used in cell bioreactors [139]. Additionally, P188 was determined to reduce endothelial adherence and improves the rheology of sickled red blood cells [140], leading to P188 in clinical trial as a therapeutic agent for sickle cell anemia [141–143]. A main outcome of a ~ 350 patient sickle cell anemia trial was its safety profile in long-term use. P188’s first FDA approved use in humans was as a skin wound cleanser that has demonstrated lack of toxicity to the cellular components of blood and lack of interference to the wound’s ability to heal and resist infection after being tested in more than 1000 patients [144, 145].

### Copolymer-based muscle membrane stabilization: cellular studies

The first applications of P188 in muscle demonstrated significant reduction in electroporation-induced leakage of carboxyfluorescein dye from isolated skeletal muscle cells [118]. In parallel experiments, the hydrophilic control molecule Dextran showed no membrane protective effect [118], suggesting that P188 interacts with the damaged membrane in a way that alters membrane properties and promotes stability. Other reports produced similar results in in vitro models of acute radiation injury which involves the generation of reactive oxygen species which can rapidly alter the structure and organization of the cell membrane leading to cell necrosis. In a study by Hannig et al. [146], P188 was shown to retard cytoplasmic calcein leakage from isolated rat skeletal muscle cells undergoing radiopermeabilization. Greenebaum et al. [147] further showed that skeletal muscle cells treated with P188 manifested enhanced viability and survival following high-dose irradiation.

Following these reports, a seminal study by Yasuda et al. [80] demonstrated that the acute application of P188 to isolated dystrophic *mdx* cardiac myocytes restored myocyte cellular compliance to wild-type levels by blocking passive stretch-mediated calcium overload. Dystrophic *mdx* cardiac myocytes demonstrated increased passive tension during extension, resulting, in part, by the influx of extracellular Ca^2+^ during physiological passive myocyte lengthening. P188 fully normalized myocyte passive compliance to normal levels [80]. At the level of the whole organ, P188 decreased passive tension and thereby improved myocardial relaxation, allowing for complete filling of the ventricles and return to normal working end diastolic and end systolic volumes [29].

### Copolymer-based membrane stabilizers in vivo

Yasuda et al. further showed that in vivo systemic administration of P188 to *mdx* mice improved ventricular geometry and prevented acute cardiac failure during a dobutamine cardiac stress test protocol [80]. In the golden retriever dystrophic canine model, chronic P188 administration prevented left-ventricular remodeling, reduced myocardial fibrosis, and blocked cardiac troponin I release [148]. In addition, long-term intermittent administration of P188 was shown to confer protection during isoproterenol-induced cardiomyopathy in *mdx* mice [149].

The ability of synthetic membrane stabilizers to protect fragile DMD skeletal muscles had, up until recently, been less clear. Early investigations with P188 showed little to no efficacy in protecting dystrophic limb skeletal muscle function in vivo [150, 151], even though P188 had been shown effective in protecting hindlimb skeletal muscle in a range of other conditions, including electrocution injury [118, 152], hindlimb ischemia-reperfusion injury [153, 154], and in a model of dysferlin-deficiency [155]. Interestingly, a recent study evaluating the pharmacodynamics of P188 demonstrated P188 can fully protect dystrophic skeletal muscle against mechanical stress in vivo [156]*.* This study showed how in vivo membrane protection is critically dependent on delivery route [156] wherein subcutaneous delivery of P188 led to dramatic improvement in *mdx* hindlimb muscle function during lengthening contractions and decreased uptake of Evans blue dye in vivo. In contrast, in this model, neither intraperitoneal nor intravenous delivery, which were routes used in previous studies, led to improvement in muscle function [156]. Thus, the lack of skeletal muscle efficacy reported in previous studies using P188 [150, 151] could be attributed to suboptimal mode of delivery of P188, rather than a fundamental limitation in the mechanism by which the block copolymer stabilizes fragile dystrophic skeletal muscle membranes. This was further supported by another recent study showing that chronic dosing of P188 using subcutaneous delivery improves diaphragm function in *mdx* and *mdx:utr*^−/−^mouse models in vivo [157]. In that study, P188 improved dystrophic mouse respiratory parameters in vivo, including tidal volume/body weight and minute volume/body weight, as well as decreased central nucleation and decreased collagen deposition in treated diaphragm muscle fibers [157]. These results are promising in indicating that chronic P188 treatment may be beneficial in preserving respiratory and limb muscle functions. Taken together, these findings are evidence that synthetic membrane stabilizers provide a unique first-in-class treatment strategy for simultaneously treating all affected striated muscles in DMD. A summary of in vivo studies testing block copolymers as a therapeutic strategy in DMD models is presented in Table 2.

**Table 2:** Summary of studies using block copolymers as a treatment in DMD models in vivo

Copolymer	Pathophysiology	DMD model	Treatment time	Dosage	Delivery route	Results	References
P188	Cardiomyopathy	*mdx*	Pre-treatment (30 min)	460 mg/kg	i.v.	P188 significantly improved cardiac hemodynamic response and animal survival during cardiac stress testing	Yasuda et al. (2005) [80]
P188	Skeletal muscle	*mdx*	Pre-treatment (30 min)	600–1800 mg/kg	i.p.	No significant difference in % EBD penetration in rectus femoris muscle fibers in P188 treated *mdx* mice exercised by downhill treadmill running	Quinlan et al. (2006) [150]
P188	Cardiomyopathy	*GRMD*	8 weeks	60 mg/kg/hr	i.v.	Chronic P188 treatment normalized serum cTnI levels, blocked increases in heart failure marker BNP, significantly decreased cardiac fibrosis, and prevented dilated cardiomyopathy. Cardiac hemodynamic function in response to dobutamine stress was significantly improved compared to saline treatment. Serum CK levels were not affected.	Townsend et al. (2010) [148]
P188	Cardiomyopathy	*mdx*	2–4 weeks	460 mg/kg	i.p.	P188 treatment prevented a decrease in cardiac function in response to isoproterenol stress testing. Treated mice did not show significant differences in cardiac fibrosis but had increase in EBD positive fibers, these hearts showed increased systolic function compared to untreated hearts.	Spurney et al. (2010) [149]
P188	Skeletal muscle	*mdx*	Pre-treatment 2-week daily	30 mg/kg, 460 mg/kg	i.p.	Single dose P188 treatment induced an increase in specific force and decreased the number of IgG positive fibers in both non-stressed and stressed muscles. P188 treatment improved the histological appearance in TA muscles under some conditions. 2-week P188 did not affect TA force. During lengthening contraction injury, it was reported that in a subset of contractions the P188 treatment group had slightly but statistically significant lower force than saline control.	Terry et al. (2014) [151]
P188, P338	Skeletal muscle	*mdx*	Pre-treatment (0.5–3 h)	60–460 mg/kg	i.p., i.v., s.c., i.m.	Subcutaneous but not intravenous nor intraperitoneal injection of P188 significantly decreased the force loss during and after lengthening contractions of hindlimb *mdx* muscle and significantly decreased EBD uptake into TA myofibers post-injury. Subcutaneous delivery of PEO8000 had no protective effect. Lower dosage of intraperitoneal and intramuscular but not subcutaneous or intravenous injections of P338 shows significant protective effect.	Houang et al.(2015) [156]
P188	Respiratory	*mdx*	Q.D., 22 weeks	3 mg/kg	s.c.	Chronic delivery of P188 had significant positive effects on respiratory function parameters and improved diaphragm histological parameters and caused improvement in cardiac hemodynamics of treated *mdx* mice	Markham et al. (2015) [217]
	Cardiomyopathy	*mdx/utr* ^*−/−*^	Q.D., 8 weeks	1 mg/kg	s.c.	P188 treatment slowed the loss of respiratory function and improved diaphragm histological parameters in double knockout mice	
diP188 diP188-CH_3_ diP188-(CH_3_)_3_	Skeletal muscle	*mdx*	Pre-treatment (0.5–3 h)	1000 mg/kg	i.p.	A diblock copolymer architecture confers membrane stabilization. The addition of a single hydrophobic tert-butoxy end-group to the PPO core significantly enhanced membrane protection against lengthening contractions. The less hydrophobic methoxy and hydrophilic hydroxyl end groups did not confer membrane protection in vivo.	Houang et al.(2017) [183]

*i.v.*, intravenous; *i.p.*, intraperitoneal; *s.c.*, subcutaneous; *i.m.*, intramuscular; *EBD*, Evans blue dye; *GRMD*, golden retriever muscular dystrophy; *cTnI*, cardiac troponin I; *BNP*, brain natriuretic peptide; *CK*, creatine kinase; *TA*, tibialis anterior; *Q.D.*, daily; *diP188*, diblock P188

### Elucidating the copolymer-muscle membrane interface

The mechanism underlying copolymer-lipid bilayer interaction has not been delineated. Elucidating copolymer chemical and structural characteristics are essential to determine membrane stabilizer function, under both normal and disease conditions. Because biological membranes are structurally complex, artificial phospholipid-based membranes are an invaluable model to study the biophysical basis of copolymer-membrane interactions. To investigate the physical nature of P188-membrane interactions, Cheng et al. employed ^1^H Overhauser dynamic nuclear polarization/Nuclear Magnetic Resonance spectroscopy to determine local hydration dynamics at the P188-lipid membrane interface [123]. The high spatial resolution afforded by this technique allows for probing the local water diffusivity in lipid bilayer systems. Here, P188 weakly adsorbed to the intact vesicle membrane surface. This was shown by membrane hydration dynamics and intra-bilayer water diffusivity, both at the membrane surface and bilayer interior. Furthermore, P188 weakly adsorbed at the membrane surface and produced no measurable changes in membrane dynamics or structure, as detected by electron paramagnetic resonance and isothermal calorimetry techniques. Collectively, this is evidence that P188 does not fully insert in the intact bilayer interior nor does it affect overall lipid packing [123].

As DMD pathophysiology is exacerbated by lengthening contractions, it is important to compare results from non-stressed membranes to mechanically stressed membranes. To mimic bilayer mechanical stress using artificial membranes in vitro, studies have used Langmuir troughs. This approach permits fine control of the surface area and therefore lipid packing density of supported phospholipid monolayers at the air/water interface [121, 158]. Maskarinec et al. [159] focused on P188 insertion as a function of surface pressure, which directly correlates to lipid packing density. Here, using either anionic dipalmitoylphosphatidylglycerol (DPPG) or zwitterionic dipalmitoylphosphatidylcholine (DPPC) monolayers, results showed P188 inserts into both lipid types at a surface pressure (π) ≤ 22 mN/m, which is lower than that of a healthy cell membrane (~ 30–35 mN/m) [160, 161]. P188 was found to remain inserted until the surface pressure increased back to threshold surface pressure equivalent to that of an intact membrane [158, 159]. X-ray reflectivity results further showed that at high surface pressure lipid films, in the presence and absence of P188 in the subphase, exhibit similar electron density profiles [121, 162].

**Fig. 3:**
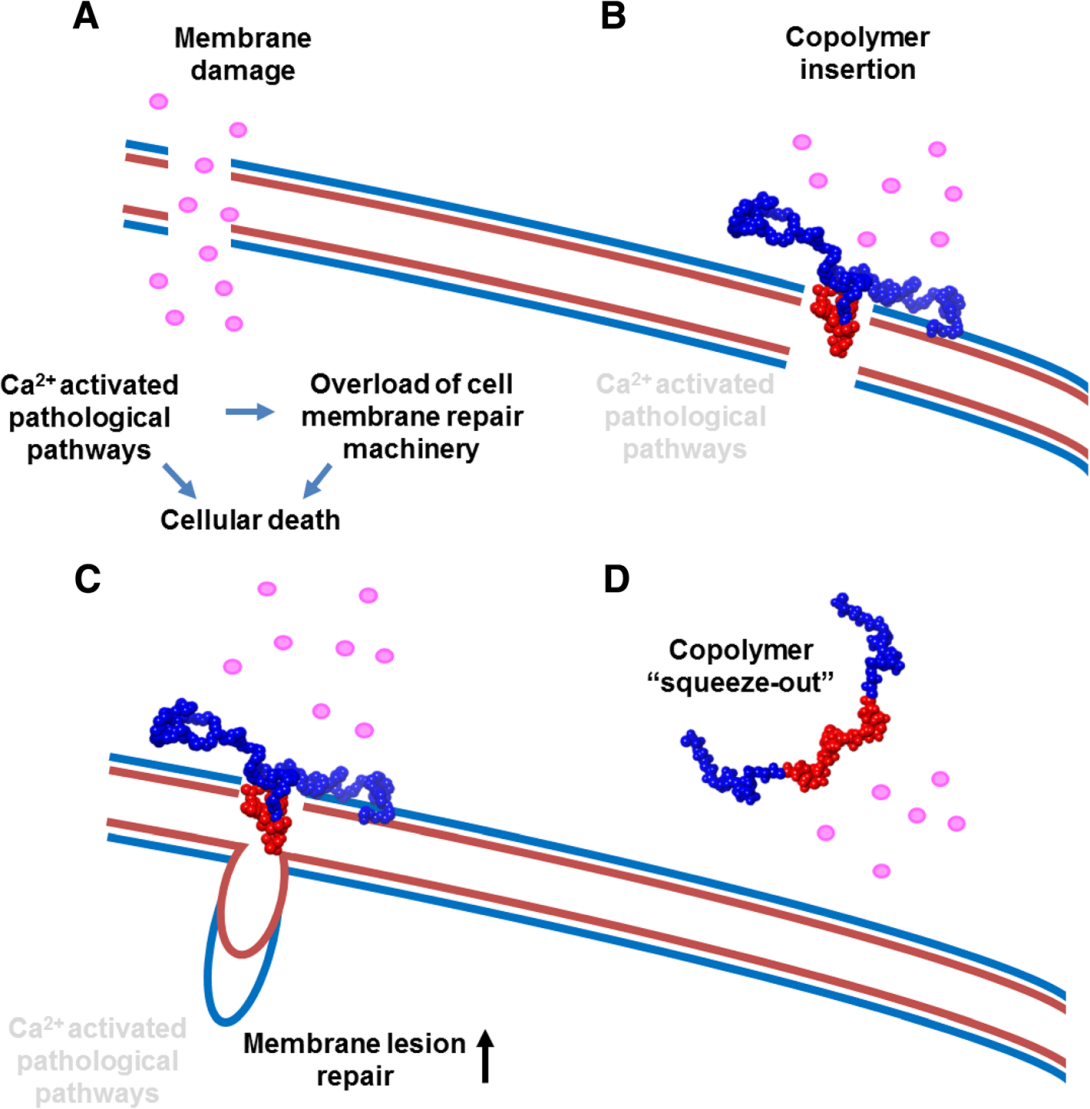
Model of copolymer-based membrane stabilization. **a** In DMD, susceptibility to sarcolemmal damage from lengthening muscle contraction renders the muscle cell membrane leaky to extracellular Ca^2+^ (pink circles). Subsequent intracellular Ca^2+^ overload leads to activation of pathological cellular pathways. Further membrane damage overloads the repair capacity of endogenous cell membrane repair mechanisms and ultimately leads to cell death. **b** Copolymer insertion driven by hydrophobic interactions (red PPO block of the copolymer with the hydrophobic part of the membrane that is now exposed due to instability). Membrane stabilization prevents pathological Ca^2+^ entry into the cell and prevents activation of cellular death pathways. **c** While the copolymer stabilizes the membrane and prevents further damage, intrinsic cell membrane repair mechanisms can repair lesions at damaged sites [215]. **d** Once the membrane integrity is restored, the copolymer membrane stabilizer is “squeezed out” of the membrane. Here, the membrane is resealed, its lipid packing density is restored, and its hydrophobic portion is now enclosed [159, 216]

Morphologically, P188 insertion appears to tighten lipid packing via physical occupation of surface area in localized patches rather than uniformly across the whole membrane [121, 159]. The hypothesis follows that only when lipid packing density is low, and the hydrophobic core of the monolayer is exposed, that P188 partitions to the membrane via hydrophobic interactions between the acyl chains of the bilayer and the copolymer hydrophobic PPO block. Inability to remain inserted above a threshold surface pressure suggests that P188 does not insert into normal intact cell membranes and only inserts once lipid density is decreased. This leads to a dynamic interaction, wherein P188 is “squeezed out” from the cell bilayer when normal membrane structure is restored (Fig. 3). Copolymer “squeeze out” upon normalization of membrane lipid packaging density is an important concept driving therapeutic applications. In this context, copolymers only insert into areas of the membrane that are damaged. This working model hypothesizes that when copolymer insertion re-establishes membrane barrier function and prevents Ca^2+^ overload during muscle contraction, the endogenous cell membrane repair response would be able to patch the membrane [1]. Upon repair, the copolymer would then disengage from the membrane (Fig. 3). This copolymer squeeze out at normal surface pressure would be beneficial in the context of biomedical applications of damaged cellular membranes where copolymers selectively insert only onto localized areas of the membrane where the local lipid density is reduced, and thus only where the membrane is structurally impaired, and not interact with intact with healthy areas of the membrane.

## Copolymer structure-function analysis

Mechanistic investigation via the structure−function relationship of block copolymer chemistry is required to define the basis of copolymer-based membrane interaction. This is crucial in the long-term to guide the design of an optimal membrane stabilizer. There is considerable interest in block copolymers as membrane stabilizers due to their overall surface active and solvent-selective characteristics and intrinsic thermodynamic properties and architectures [163, 164]. P188 is part of a large family of poloxamers, each with distinct physicochemical properties. Polyethylene glycol (PEO or PEG), the hydrophilic constituent of poloxamers, has been well investigated in the fusion of model membranes and for its ability to lower water molecule activity at the membrane-solvent interface [165]. While PEO-mediated membrane stabilization has been shown to be effective, the very high concentrations (mM-M) required for effectiveness indicate that the hydrophobic block plays an essential role in copolymer-membrane interactions [166].

The relationship between copolymer chemical structure and the kinetics of adsorption, insertion, and subsequent squeeze out from lipid monolayers has been investigated by Frey et al. via Langmuir trough experiments and Monte Carlo simulations [120]. Here, upon compression of the monolayer, copolymers with higher PPO/PEO ratio favored a higher squeeze out pressure. Moreover, higher molecular weight copolymers were observed to squeeze out at higher surface pressures, while at constant PPO/PEO ratios smaller copolymers squeezed out at lower pressures. Results showed that the ratio dictates the equilibrium spreading pressure of copolymers at the phospholipid interface. Hydrophobic copolymers were less soluble resulting in a higher proportion of adsorption at the monolayer interface and thus higher equilibrium spreading pressure [120]. These findings demonstrate the relationship between the PPO/PEO ratio and molecular weight in determining copolymer-membrane interactions.

Overall, copolymer hydrophobicity has a principal role in affecting membrane bilayer physical structure. Thus, more hydrophobic copolymers decrease membrane microviscosity [117, 167] and increase the rate of lipid motion across the outer and inner leaflets of vesicular membranes [117], causing membrane leakiness [115, 168]. Chang et al. [169] showed that surface pressure-area isotherms exhibited by P188 (PEO_75_–PPO_30_–PEO_75_) compared to the highly hydrophobic P181 (PEO_2_–PPO_30_–PEO_2_) are significantly different. P181 exhibits condensed-film-like surface behavior whereas P188 exhibits an expanded-like behavior. This was confirmed by Cheng et al. [123] using dynamic light scattering, isothermal calorimetry, and small molecule-directed lipid peroxidation of liposomes. The PPO/PEO ratio was shown to be a key feature in effectively protecting intact liposomes from peroxidation. Copolymers that adsorb at the membrane surface, without penetration into the bilayer core, such as P188 and PEG8000, presumably affect the hydration shell of the bilayer. This would suppress the diffusion of the free radical lipid peroxidation initiator into the lipid bilayer, thereby preventing the initiation of lipid peroxidation. The more hydrophobic poloxamers, for example, P335 (PEO_38_–PPO_54_–PEO_38_), P333 (PEO_20_–PPO_54_–PEO_20_), and P181 (PEO_2_–PPO_30_–PEO_2_), have significant heat of partitioning indicative of insertion into the liposomal membrane [123]. These hydrophobic copolymers do not prevent initiation of lipid peroxidation [170] indicating that copolymer hydrophobicity affects kinetics of insertion. More hydrophobic copolymers insert at faster rates by initially embedding below the lipid head group region, opening up the packing of acyl chains and accelerating the passage of water across the membrane, thus increasing permeability [123, 166].

**Fig. 4:**
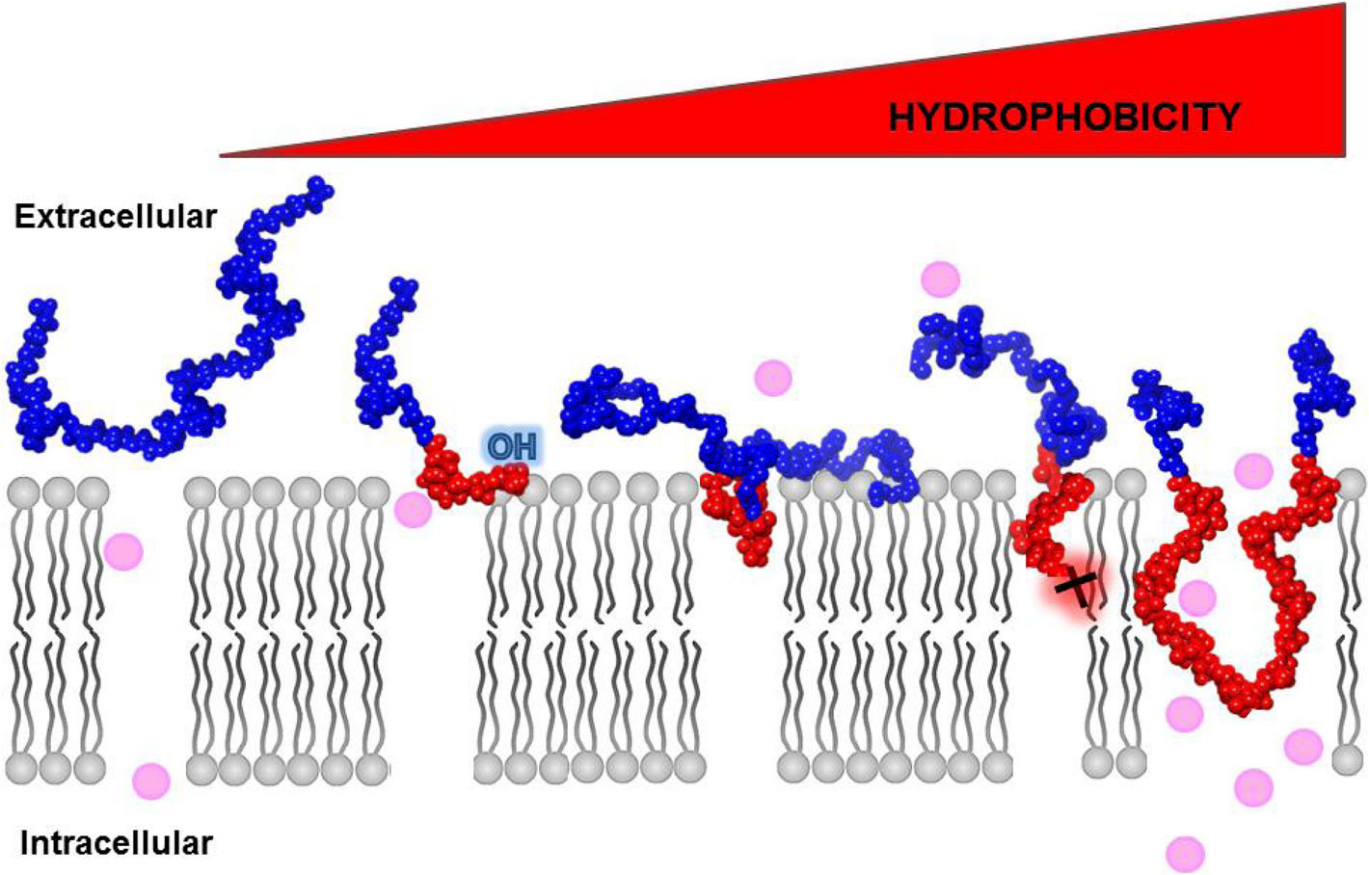
Schematic representation of structure-function of copolymer-membrane interaction. Triblock copolymer membrane stabilization occurs via insertion of the hydrophobic PPO core block (red) and balanced by flanking of the two hydrophilic PEO blocks (blue) that are required to prevent complete translocation across the membrane. Without a second flanking PEO chain, diblock copolymers can also insert into the membrane, but insertion is at least in part dictated by the PPO end group. Here, the more hydrophobic end group, such as –C(CH_3_)_3_ (†), driving insertion and anchoring and the more hydrophilic end groups, such as –OH, retained at the solvent-polar head group interface. Variation in PEO (blue) and PPO (red) block lengths alters the hydrophobic/hydrophilic balance that is required for optimal membrane insertion and stabilization. Too high a PPO/PEO ratio and large size PPO group drives the copolymer deeper into the membrane and further exacerbates damage to the membrane

The size of the hydrophobic PPO block influences insertion of the copolymer into lipid films. Poloxamers at fixed 80% PEO composition and different molecular weights (P108, P238, P188, and P338) have been tested for their relative ability to insert into lipid monolayers [158]. Copolymers with high PPO content required lower surface pressure for insertion. Additionally, once inserted, high mass copolymers are able to retain position within the monolayer at much higher surface pressures before being squeezed out [120, 158]. Moreover, hydrophobic copolymers with bulkier PPO blocks were found to increase flippase activity compared to copolymers with shorter PPO blocks [117]. Copolymer-bilayer interactions have been investigated using pulse field gradient nuclear magnetic resonance to quantify copolymer diffusion in the presence and absence of unilamellar liposomes [171]. Here, the binding percentage of copolymers to liposomes was quantified, and results further confirmed that increased copolymer molecular weight and increased relative hydrophobicity cause increased binding and liposome coverage relative to smaller, more hydrophilic copolymers. Another recent study using surface plasmon resonance to probe and compare binding of P188 and a PEO homopolymer of similar size provides direct evidence of binding onto supported intact lipid bilayers with comparable binding kinetics. Moreover, this study provides biophysical evidence that copolymer adsorption alone does not fully account for membrane protection efficacy. [172] A schematic summary of structure-function of copolymer-based membrane stabilization is presented in (Fig. 4**)**.

## Molecular dynamics analysis of copolymer-membrane interactions

Mechanistic insights into copolymer-membrane interaction are aided by studies pursued at the atomistic level. Molecular dynamics (MD) simulations have been recently developed to investigate copolymer-phospholipid bilayer interactions [173, 174]. MD simulations are physics-based computational methods to simulate and observe the interactions of atoms and molecules at resolutions that are currently hard or impossible to probe experimentally. In general, MD simulations of large macromolecules, such as copolymers, are computationally challenging to perform. Past MD efforts have focused on coarse-grained [120, 175, 176] and united atom [168, 177, 178] models, which are models that reduce the total number of degrees of freedom in the system by representing molecules and their interactions at lower resolution. This allows for significantly increased simulation timescale at lower computational cost but in exchange for the loss of atomistic level details.

An in silico model of copolymer adsorption using coarse-grained force field showed copolymer-membrane insertion, followed by percolation across the unstressed lipid bilayers [179]. Here, copolymers containing a PPO block with a length comparable to that of the bilayer thickness tended to span across, or percolate across, the lipid bilayer. In comparison, copolymers with shorter PPO blocks inserted partially, with the PEO blocks remaining in water on one side of the bilayer. Moreover, total percolation of copolymers across the bilayer led to reduction in membrane thickness and an increase in the area per lipid. Goliaei et al. [177] used an united-atom force field-based MD model to show that P188 can passively insert into the 1,2-dilauroyl-sn-glycero-3-phosphocholine (DLPC) lipid bilayer under non-stressed conditions after extensive simulation time (> 500 ns). Here, the PPO block inserted into the hydrophobic part of the bilayer and the PEO chains remained solvated outside the membrane [177]. Moreover, using a 3 nm water pore model to simulate a damaged lipid bilayer, the PPO block of P188 inserted adjacent to the water pore and “pushed” water molecules out of the pore to reduce pore size.

Simplified force field models allow for larger timescale simulation; however, they yield only a partial view of membrane structural properties and limit atomic resolution insights [180]. Importantly, previous MD studies have focused on copolymer-bilayer interactions under constant pressure and temperature (NPT) and constant area and temperature conditions (NPAT), and thus are computational models of membranes under normal non-stressed conditions. Recently, an all-atom MD simulation model was developed to investigate copolymer-lipid membrane interaction under conditions of varied lateral mechanical stress. This in silico approach correlates to the physiological state to lengthening contraction muscle injury in DMD. Here, an increase in surface tension (γ) was applied to induce expansion in the bilayer area per lipid molecule (*A*_0_) to model bilayer mechanical stress [181]. P188 interaction with lipid bilayers was demonstrated to be dependent on *A*_0,_ with insertion of the PPO block occurring at a ~ 15–20% increase in *A*_0._ Additionally, P188 insertion into the membrane significantly increased the lateral pressure required for membrane rupture under mechanical stress [181]. Further, membrane insertion and stabilization efficacy appeared dependent on the PPO/PEO ratio. MD simulations of hydrophobic copolymers, such as P331 (PEO_7_–PPO_54_–PEO_7_), inserted at significantly lower *A*_0_, as well as decreased the lateral pressure required to rupture the membrane. This is consistent with the results of Nawaz et al. [168] who demonstrated percolation across the bilayer of highly hydrophobic copolymers causing membrane bending and an increase in local permeability allowing water molecule penetration into the hydrophobic region of the membrane. The timescale for percolation was inversely proportional to the PEO block length [168]. Moreover, another all-atom MD study by Zaki and Carbone showed that incorporation of multiple copolymer units within the bilayer hinders lipid diffusion and forced nearby lipids to remain closely packed, even during lateral mechanical stress [182].

Overall, the results from MD studies are consistent with experimental observations from Langmuir trough studies in that P188 inserts into areas of low lipid density and at low surface pressures [158, 159]. MD studies feature a simplified phospholipid bilayer as a basic model of the biological membrane, which is comprised of proteins, complex mixtures of lipid types, and other macromolecules, all organized in a tightly regulated manner. Nonetheless, all atom MD results are qualitatively comparable to results derived in cells and animals [156, 183, 184]. Complementation of findings from in silico to in vivo methods underscores MD simulations as a powerful tool to further mechanistic understanding of copolymer-bilayer interactions and to ultimately guide design and optimization of copolymers for physiological membrane stabilization.

## Copolymer architecture: diblock copolymers as membrane stabilizers

Block copolymers can be designed with two or more distinct polymer blocks covalently bonded together. These can exist in a variety of molecular sizes, relative degree of polymerization of each block (composition), hydrophobicity, chemical moieties, and architectures, from diblock and triblock to multi-blocks. This broad landscape leads to a nearly infinite number of possible distinct chemical configurations [112]. Previously, from a practical perspective, the use of poloxamers has been generally constrained to those made available commercially. This limitation provides an impetus for advancing discovery of the copolymer chemical landscape beyond that of the triblock architecture. As above, P188 is reported to be weakly adsorbed to the lipid bilayer [123, 170] and it is hypothesized that this weak association is due to steric constraints imposed by the flanking PEO chains [162]. The removal of one of the flanking PEO chains to form the diblock PEO–PPO architecture (Fig. 2) allows for facile assessment of the association of the hydrophobic PPO core with the lipid bilayer.

Firestone et al. employed small- and wide-angle X-ray scattering techniques to examine the structure of a lipid bilayer and the phase produced by either the triblock P188 or a PEO–PPO diblock with an equivalent PPO block length [185]. P188-synthetic lipid bilayer interaction produced an aggregate phase structure suggesting limited insertion of the copolymer into the lipid bilayer. On the other hand, the PEO–PPO diblock produced a well-ordered lamellar phase suggesting enhanced interfacing within the bilayer [185]. This suggests that removing one of the flanking PEO chains facilitates PPO block interaction with the hydrophobic acyl chain region of the lipid bilayer to strengthen copolymer-bilayer interaction.

The PEO–PPO diblock architecture offers several advantages for advancing copolymer-membrane structure-function studies. These include an easier and more controlled chemical synthetic process [186], the more precise control of PPO and PEO block sizes and the ability to design specific functional end groups to the hydrophobic PPO core to finely tune membrane interactions. This latter modification allows for sensitive modulation of the diblock PPO block hydrophobicity. This strategy has precedence in the surfactancy literature where novel terminal functional groups have been shown to influence solution and bulk phase behavior [187, 188]. Diblock copolymers have never been investigated for biological membrane stabilization until a recent report demonstrating that diblock PEO–PPO architectures can confer membrane stabilization in both in vitro and in vivo DMD models [156, 171, 183, 184]. This establishes that specific PPO end group chemistries play a critical role in defining muscle membrane stabilization [183, 184].

Recent diblock studies have advanced an “anchor and chain” model of membrane stabilization (Fig. 4) [156, 171, 183, 184]. Here, the addition of a small hydrophobic end group “anchor,” as demonstrated by tert-butoxy to the PPO block, discretely increases the hydrophobic character of the end of the PPO block, without significantly increasing the overall mass of the copolymer. From these results, it is hypothesized that discrete alterations in the structure of the PPO terminal functional group, such as replacing *tert*-butoxy with *n*-butoxy or other non-polar end groups, will further influence the packing and interaction strength with the lipid core. The PEO chain appears to be required to preserve the amphiphilic behavior of the copolymer and to maintain the copolymer at the solvent-membrane interface. Detailed structure-function analysis of the PEO block, including length, structure, and chemical characteristics, has not yet been initiated and this will be important to determine in further experimentation. Taken together, these proof-of-principle results establish physiological relevance to diblock copolymers and support further investigation of this expansive copolymer chemical space.

## Clinical applications, challenges, and ongoing developments

P188 was first approved by the FDA as an anti-viscosity agent added to blood before transfusions [135, 189]. P188 (labeled as RheothRx, Glaxo Wellcome Inc.) has been previously tested in clinical trials for both sickle cell anemia [141, 142] and myocardial infarction [190, 191]. Due to its nature as a nonionic surfactant and demonstrated hemorrheologic properties, a randomized, double-blind, placebo-controlled pilot study in 50 patients was initiated in the early 1990s to determine the safety and efficacy of P188 in treating acute vaso-occlusive crises in sickle cell anemia disease. Treated patients showed a significant decrease in painful episodes, reduced hospital stay, requirement of analgesics, and reported pain [142]. Moreover, continuous RheothRx intravenous infusion over 48 h (60-min loading dose of 300 mg/kg followed by a 47-h maintenance infusion of 30 mg/kg) was well tolerated with the exception of a mild increase in serum creatinine in one patient with underlying renal dysfunction.

Pharmacokinetic study of P188 injection in healthy males has been conducted in a cohort of volunteers and determined that elimination occurs primarily through renal clearance [192]. RheothRx (P188) clinical trial in patients tested adjunctive therapy during thrombolytic therapy for acute myocardial infarction at time of hospitalization. Initial reports showed P188 resulted in significantly smaller-sized infarcts, greater myocardial salvage, and improved median ejection fraction [191]. However, in follow up large-scale clinical studies, Rheothrx administration did not significantly decrease infarct size or favorably alter outcome [193]. Moreover, in a subset of elderly patients with pre-existing renal disease increased renal dysfunction was reported. This adverse effect was later determined to be due to small molecular weight impurities in the P188 formulation, which was manufactured as an excipient-grade product following National Formulary specifications [194]. Subsequent clinical studies using the purified formulation of P188 significantly improved the renal safety profile and tolerability [194].

Purified formulation of P188 was repackaged as MST-188 or vepoloxamer (Mast Therapeutics, Inc.) which was then further evaluated in another interventional clinical trial (EPIC trial) in children with sickle cell disease. In a recent large-scale phase 3 clinical trial, vepoloxamer did not meet primary efficacy endpoints of demonstrating a statistically significant reduction in the mean duration of vaso-occlusive crisis events. However, this clinical trial did show that vepoloxamer was generally well tolerated with no statistically significant differences in treatment-related adverse events in the vepoloxamer group compared to the placebo group (https://clinicaltrials.gov/ct2/show/NCT01737814).

For membrane stabilizers in DMD, Phrixus Pharmaceuticals, Inc. has initiated a Phase 2 single site, open-label trial for respiratory, cardiac and skeletal limb muscle end points in non-ambulatory DMD patients (ClinicalTrials.gov Identifier: NCT03558958). Drug P-188 NF (Carmeseal-MD) is directed toward DMD patients with early heart failure and respiratory dysfunction who are currently on stable regimen of background therapies. Phrixus Pharmaceuticals and Ethicor Pharma Ltd. have made Carmeseal-MD available in 2015 as a “special” or unlicensed medicinal product in the European Union prior to regulatory approval. This allows access to Carmeseal-MD to DMD patients with respiratory and cardiac deficits through physician request. As of the end of 2017, one patient under the Expanded Access Program has been reported to have met the 15-month treatment mark with treatment reported to have been well tolerated and reductions in creatine kinase and cardiac troponin I observed (Phrixus Pharmaceuticals). Moving forward, larger scale human clinical data will be required to fully evaluate membrane stabilizer treatment efficacy in DMD patients.

## Conclusions

From a conceptual perspective for clinical application, synthetic muscle membrane stabilizers for treating DMD patients have several attractive features. These include (1) treatment strategy targeting the primary defect in DMD—severe muscle membrane instability causing muscle deterioration and cell death, (2) copolymers as muscle membrane interfacing molecules could in principle treat all DMD patients regardless of their genetic lesion, (3) pre-clinical studies provide evidence of copolymer protection in other applications, (4) first-in-class membrane stabilizer P188 NF has a favorable safety profile in cardiac, respiratory, and limb striated muscles, as derived from human clinical trial data in humans. The inherent limitation with membrane stabilizers as a potential therapy for DMD is that this approach is not a cure and would necessitate chronic treatment for DMD patients.

The ultimate goal for membrane stabilizing therapy is to significantly improve and prolong patient quality of life while awaiting a potential effective cure for DMD. As DMD is a chronic progressive disease, membrane stabilization treatment would require life-long administration. In the best case scenario, this clinical treatment would effectively manage the disease, analogous, for example, to the highly effective life-long daily insulin treatment used by type I diabetic patients. One could envision chronic copolymer treatment starting soon after diagnosis with the aim to preserve striated muscle function before muscle degeneration and wasting occurs. Membrane stabilizers may also be envisioned in acute settings for DMD patients, for example, during orthopedic surgery or other stress-inducing events [148]. Another setting where copolymer administration could make a significant positive impact is during exercise training protocols for DMD patients implemented to oppose the loss of functional abilities as a result of muscle disuse [195]. It is still unclear whether exercise training and which exercise protocols could be beneficial to DMD patients or other patients with myopathic disorders, at least in part due to the potential detrimental effects of strenuous exercise and muscle contraction on the muscle membrane [196]. Treating DMD patients with membrane stabilizers prior to an exercise training bout may support striated muscle membranes during strength exercise and abrogate deleterious effects that would occur while supporting muscle repair and strength building.

It is also likely that effective DMD treatment will ultimately require a combination of approaches to achieve optimal outcomes. One example where bundled therapies containing P188 has already shown promise is cardiac arrest and resuscitation [197]. Block copolymers have been in use as vehicles for enhanced gene delivery in other applications [198, 199], and the prospect of bundled therapies of block copolymers and gene-directed strategies would be of significant interest to pursue in future works. Another strategy where copolymer-based membrane stabilizers could be combined would be stem cell therapy to regenerate muscle. Induced pluripotent stem cell (iPSC) technology allows derivation of patient-derived stem cells which obviates immunological concerns. One recent study showed proof-of-principle application of ex vivo genetic correction of dystrophic iPS cells with a micro-utrophin transgene before transplantation back into dystrophin/utrophin double knockout mice [200]. They observed that engrafted muscle had large numbers of corrected myofibers, restoration of the dystrophin and associated proteins complex and improved contractile strength. While these results are positive and exciting, this strategy still has to overcome multiple important hurdles, such as improved survival of the cells post-injection, effective migration to the compromised muscles, and successful engraftment. Copolymer-based membrane stabilizers injected alongside iPS-derived myocytes may help improve survival of these cells post-injection.

Synthetic membrane stabilizers may ultimately extend to numerous other inherited or acquired diseases in which cell membrane integrity is compromised. In the last few years, many preclinical studies using P188 as cell membrane stabilizers have been published in a variety of pathological settings, including amyotrophic lateral sclerosis [201], traumatic brain injury [202], aggregation of unfolded protein [203–207], hypoxia and ischemia-reperfusion injury [154, 208, 209], irradiation and burn injury [152, 210, 211], cartilage damage, and joint degeneration following blunt impact [212–214]. Based on the potential novel uses of copolymer-based membrane stabilizers in various other diseases where the cell membrane is damaged, one could anticipate that increased academic and clinical interest in this therapeutic strategy will help promote faster translation to human clinical applications.

Finally, as detailed in this review, first-in-class copolymer-based membrane stabilizer P188 has a long history. Developed over 70 years ago for industrial applications, it is now clear that P188 has unique properties enabling its interfacing with lipid bilayers, including damaged muscle membranes. In this context, significant opportunities for advancing copolymers in biomedical applications are apparent. Detailed copolymer structure-function studies, which will require concerted trans-discipline collaborations between copolymer chemists, chemical engineers, molecular and integrative physiologists, and clinicians, can be expected to provide new insights into the mechanism by which copolymers interface with damaged muscle membranes. Armed with new structure-function insights, one could envision precise refinements in copolymer design to enhance muscle membrane stabilizer efficacy/duration-of-action for treating devastating diseases, including DMD.

## Abbreviations

DGC: Dystrophin-glycoprotein complex
DLPC: 1,2-dilauroyl-sn-glycero-3-phosphocholine
DMD: Duchenne muscular dystrophy
DPPC: Dipalmitoylphosphatidylcholine
DPPG: Dipalmitoylphosphatidylglycerol
MD: Molecular dynamics
NPAT: Constant pressure, area, and temperature
NPT: Constant pressure and temperature
NPγT: Constant pressure, surface tension, and temperature
PEG: Polyethylene glycol
PEO: Polyethylene oxide
PPO: Polypropylene oxide
ROS: Reactive oxygen species
